# Consumer mobility and shopping mall visitation data from smartphone tracking in Johannesburg, South Africa

**DOI:** 10.1016/j.dib.2026.112648

**Published:** 2026-03-02

**Authors:** Koech Cheruiyot, John Karuitha, Yewande Adewunmi, Prisca Simbanegavi, Kalasipa Moenyane, Kelly Cohen

**Affiliations:** a University of the Witwatersrand, School of Construction Economics and Management, 1 Jan Smuts Avenue, Braamfontein, Johannesburg 2000, South Africa; b Department of Business and Economics, Karatina University, P.O. Box 1957-10101, Karatina, Kenya; c Fetch Analytics, 11 9th Street, Houghton Estate, 2196 Johannesburg, Gauteng, South Africa

**Keywords:** Urban mobility, Retail analytics, Consumer segmentation, Geospatial data, Socio-economic behaviour

## Abstract

This dataset, sourced from Fetch Analytics, captures observations of consumer visits (*n* = 87,204,056) to three major shopping malls in Johannesburg, South Africa, from January 2022 to December 2023. The dataset collected using smartphone signal tracking provides insights into consumer behaviour. Key variables include mall name, visit frequency, distance travelled from origin (home) to destination (mall), and socio-demographic indicators such as gender, age, income, and Living Standard Measure (LSM). The dataset allows for granular analyses of how spatial and socio-economic factors influence shopping patterns in a fragmented retail landscape. This dataset is valuable for researchers investigating consumer behaviour, spatial economics, and urban retail planning.

Specifications Table

**Table:** 

Subject	Social Sciences
Specific subject area	Urban Studies, Economic Geography, Consumer Psychology, Retail Analytics
Type of data	CSV XLSX
Data collection	The dataset is collected using **smartphone signal tracking** through Fetch Analytics’ proprietary platform [6]. This technology captures real-time consumer movements to and from **three major malls** in Johannesburg: Sandton City, Mall of Africa, and Fourways Mall. To ensure **privacy compliance**, all data is anonymized, removing any personally identifiable information while preserving valuable insights into shopping behaviour [1].
Data source location	– The data was collected in Johannesburg, South Africa in three shopping malls: – Mall of Africa (-26.0148, 28.1065) – Fourways Mall (-26.0189, 28.0064) – Sandton City Mall (-26.10833, 28.05417) – The data is available on the link https://data.mendeley.com/datasets/pzpnbk5xz2/5. Visit Fetch Analytics at https://fetchanalytics.ai/ for more recent and extensive data available with upon subscription [7].
Data accessibility	Repository name: Data identification number: 10.17632/pzpnbk5xz2.5 Direct URL to data:https://data.mendeley.com/datasets/pzpnbk5xz2/5.Instructions for accessing these data: 1. **Visit the Mendeley Website**: Go to https://data.mendeley.com/datasets/pzpnbk5xz2/5 for access to the data. 2. **Download Files**: Download the following key files: a. **MetroData.csv** – General data about Johannesburg, including demographics and infrastructure. b. **Mobility and Psychographics Data** – Shopper movement and behavioural insights for Sandton City, Mall of Africa, and Fourways Mall. c. **Shoppers Journey Data** – Records distances covered by shoppers traveling to and from malls. d. **fully_joined_data.csv** – A merged dataset combining all the above files for comprehensive analysis. e. **Data format 20240407.xlsx** that describes the fields and the associated data tables. f. Maps_johannesburg.mpkx a map file that contains relative locations of the malls.
Related research article	

## 1 Value of the Data

• This dataset provides high-resolution, real-world consumer mobility data that can be used to study shopping behavior within a historically segregated and spatially unequal urban retail system, offering empirical grounding for research in urban economics, economic geography, and retail studies [2].
• The data enables statistical and econometric analysis of how distance, socio-economic characteristics (e.g., Living Standard Measure, inferred income, age cohorts), and accessibility influence consumer decisions regarding where and how often to shop, making it suitable for both exploratory and inferential research [3].
• Urban planners, retail developers, and policymakers can use the dataset to assess mall catchment areas, consumer reach, and transport-related accessibility, supporting evidence-based decisions on retail location planning and infrastructure investment [4].
• The dataset is particularly valuable for statistics, econometrics, and data science education. Statistics lecturers can use it as a teaching dataset for:
  ○ Descriptive statistics and data visualization (e.g., distributions of travel distances and visit frequencies),
  ○ Hypothesis testing (e.g., differences in visitation patterns across income or LSM groups),
  ○ Regression analyses (e.g., modeling visit frequency as a function of distance and socio-economic characteristics),
  ○ Spatial analysis and introductory geospatial statistics.
• Because the data are large-scale, anonymized, and derived from real-world smartphone signals, they are well suited for hands-on coursework, student projects, and postgraduate training, allowing learners to engage with authentic data that reflect actual urban and consumer dynamics rather than simulated examples [5].
• The dataset also supports predictive modeling and machine learning applications, such as customer segmentation, demand forecasting, and clustering of mall catchment areas, making it relevant for advanced analytics courses and interdisciplinary research [9].

## 2 Background

Retail activity in Johannesburg is deeply influenced by socio-economic disparities and urban mobility patterns. Shopping malls function as major commercial hubs, attracting consumers from diverse income groups and geographic areas. Understanding how shoppers move within these spaces provides crucial insights into purchasing behavior, spatial accessibility, and market segmentation [3].

This dataset was sourced free of charge with permission from Fetch Analytics (more details on the site https://fetchanalytics.ai/), a platform that provides consumer mobility data to help businesses analyze customer visit patterns, time spent in stores, and comparisons with competitors or branches. More extensive and updated datasets are available through their platform for a fee [7].

The dataset leverages on smartphone signal-tracking technology to capture real-time mall visits, enabling an in-depth analysis of foot traffic trends [8]. Additionally, socio-demographic indicators, such as Living Standard Measures (LSM) and inferred income levels, provide valuable insights applicable to urban planning, retail investment, and consumer behavior research [5].

## 3 Data Description

The dataset consists of multiple files that provide comprehensive insights into consumer mobility and psychographics. All the data is in the folder labelled “data” [4]. The folder has several data files.

1. “Metro Data 2024.csv”: Contains general data about the city of Johannesburg, South Africa, including demographic, economic, and infrastructure-related attributes.
2. “Mobility_and_Pyschographics Fourways.xlsx”: Includes detailed consumer movement and behavioral insights for Fourways Mall. This data captures visit frequency, shopper demographics, and consumer journey patterns to and from the malls.
3. “Mobility_and_Pyschographics Mall of Africa.xlsx”: Includes detailed consumer movement and behavioral insights for Mall of Africa. This data captures visit frequency, shopper demographics, and consumer journey patterns to and from the malls.
4. “Mobility_and_Pyschographics SandtonCity.xlsx”: Includes detailed consumer movement and behavioral insights for Sandton City. This data captures visit frequency, shopper demographics, and consumer journey patterns to and from the malls.
5. “Shopper Journey _To_And_From_Mall Fourways Mall.xlsx”: Records the distances covered by shoppers traveling to and from the Fourways Shopping mall, offering insights into mobility patterns and the geographical reach of each retail center.
6. The fully_joined_data.xlsx file is a merged dataset combining all the above information into a single file, facilitating integrated analysis of urban mobility and shopping behavior.
7. Finally, there is also a data dictionary “Data format 20,240,407.xlsx” that describes each of the fields and the associated data tables.

These files enable researchers to explore mall visitation trends, socio-demographic influences, and spatial consumer behaviors with an elevated level of granularity. Table 1.1 summaries the variables contained in the file fully_joined_data.xlsx.

**Table 1.1:** Data structure overview of the aggregated dataset.

Variable Group	Variable Name(s)	Description	Data Type / Unit
Identifiers & Ranking	rank	Relative rank of origin areas based on aggregated day visits (lower rank = higher visitation)	Integer
	pid	Unique identifier for the shopping mall	Integer
	name	Name of the shopping mall	Categorical
Spatial Attributes	Catchment (also suburb)	Unique identifier for geographic origin area	Integer
	catchment_name	Name of the origin catchment area	Categorical
Temporal Coverage	year	Year of observation	Integer (YYYY)
Mobility Intensity	number_of_visits	Total observed visits from origin to mall (annual aggregate)	Integer
Mobility Distance	distance_km	Average distance travelled from origin to mall	Numeric (km)
Age Composition	x15_19_years – x75_and_above	Proportion of visitors by age cohort.	Numeric (0–1)
Housing Type	Multiple variables (From House cluster/townhouse to Caravan)	Proportion of mall visitors by residential housing type.	Numeric (0–1)
Lifestyle Segments	Multiple variables (from Student/scholars to heavy hitters)	Proportion of visitors by socio-economic lifestyle.	Numeric (1–10)
Gender Composition	male, female	Proportion of visitors by gender.	Numeric (0–1)
Education Levels	Multiple variables (from No schooling to postgraduate degree)	Proportion of visitors by highest educational attainment categories.	Numeric (0–1)
Household Life Stage	Multiple variables (At home singles to mature Family)	Proportion of visitors by Household lifecycle stage composition.	Numeric (0–1)
LSM Segments	LSM level 1 through Level 10	Proportion of visitors in each LSM segment	Numeric (0–1)
Transport Mode	Multiple variables (From taxi to train- Gautrain)	Proportion of visitors by primary mode of transport to mall.	Numeric (0–1)
Income Bands	Multiple variables (from 0–1999 Rands to 60,000 Rands and above)	Proportion of visitors by income categories (currency: SA Rand).	Numeric (0–1)
Aggregated Indicators	Multiple variables	Aggregated age, housing, education, transport, LSM and income indicators.	Numeric (0–1)

Notes:.

1. All proportional variables represent aggregated shares of visitors from each catchment area and sum to one within their respective categories, subject to rounding.

2. Aggregated Indicators: We construct the following aggregate indicators based on the primary variables.

a. **Age**: Youth (18–35), Working Age (35–60), Pensioners (60+).

b. **Housing**: Formal or informal.

c. **Education**: Primary, Secondary, or College.

d. **Transport**: Public or Private Transport.

e. **LSM**: Low, Medium, or High LSM.

f. **Income**: Low (below 50,000 Rands per month) or high income.

Source: Authors’ construction.

To provide an overview of the dataset and to facilitate the assessment of potential biases, descriptive statistics are reported for two core variables that capture the scale and spatial structure of consumer mobility: the total number of mall visits and the distance travelled (in kilometers) from origin to destination.

Given the large dimensionality of the dataset (101 variables), these variables were selected because they are observed across all records, are central to the construction of the dataset, and are directly informative of coverage, intensity, and spatial reach, which are critical for identifying sampling and measurement biases in mobility data.

Descriptive statistics are computed for the entire population of observations, grouped by year, allowing readers to assess temporal consistency, distributional properties, and potential distortions arising from smartphone-based data collection (e.g., overrepresentation of short-distance trips or frequent visitors).

Summary measures, including the mean, dispersion, and distributional quantiles, indicate the presence of expected right-skewness in travel distances and visit frequencies, which is characteristic of large-scale urban mobility datasets (see Table 1.2). These statistics provide transparency regarding the structure of the data and support informed interpretation in downstream analyses.

**Table 1.2:** Descriptive statistics of mall visits.

**Variable**	**Year**	**Mean**	**SD**	**Min**	**Q1**	**Median**	**Q3**	**Max**
Visits	2022	9974.57	31,941.20	546.00	1463.00	2739.00	6534.00	651,285.00
Visits	2023	12,879.94	41,091.50	738.00	1835.50	3403.00	8652.50	830,166.00
Distance	2022	186.48	314.87	0.65	17.81	34.88	231.49	1299.82
Distance	2023	161.15	304.02	0.65	16.27	30.34	104.15	1296.30

Notes: Statistics computed from anonymized smartphone mobility data for Visit frequency and travel distance (km).

Source: Authors’ Construction.

**Fig. 1:**
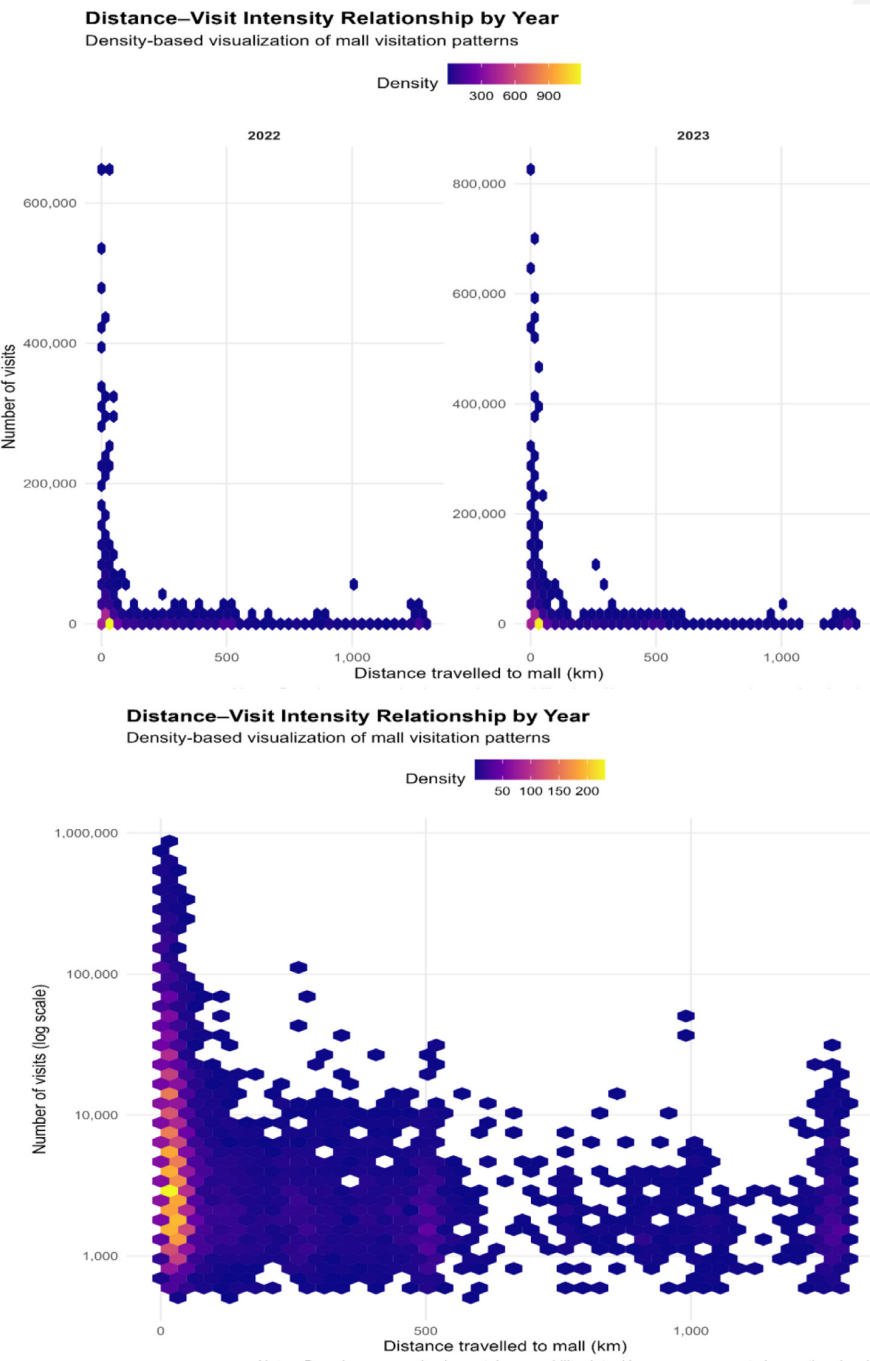
Distance-visit relationship by year. source: authors’ construction.

Fig. 1 illustrates the relationship between travel distance to the mall and visitation intensity, with the number of visits presented on a logarithmic scale to accommodate the wide variation in visit counts. The distribution shows a pronounced concentration of visits at shorter distances, consistent with distance-decay effects in urban retail mobility. Notably, dispersion in visit intensity is greatest at lower distances, reflecting substantial heterogeneity in shopping behaviour among nearby catchment areas, while long-distance trips occur less frequently and with lower variability.

In the Mobility_and_Pyschographics data files, the variable Rank refers to the relative ordering of geographic origin areas based on the total number of observed day visits to Fourways Mall, Mall of Africa, and Sandton City during the specified observation period. A lower rank value indicates a higher volume of visits originating from that area. Rankings are computed using aggregated daily visit counts and are intended to reflect the relative importance of origin zones in contributing to mall foot traffic. Life stage composition refers to the categorization of consumers into broad demographic and household lifecycle groups inferred from anonymized behavioral and location-based indicators. These groups typically reflect stages, such as youth, working-age adults, family-forming households, and older or retired individuals.

Segmentation classes represent socio-economic groupings derived from aggregated demographic, residential, and consumption-related indicators. In this dataset, segmentation includes variables, such as Living Standard Measure (LSM), inferred income brackets, and age cohorts, which are used to characterize consumer heterogeneity and shopping behavior patterns. All segmentation variables are inferred at an aggregated level and do not rely on self-reported personal information.

## 4 Experimental Design, Materials and Methods

This dataset was sourced free of charge with permission from Fetch Analytics (https://fetchanalytics.ai/) [7]. Fetch Analytics is a location-intelligence firm that provides insights into human mobility and foot traffic patterns using anonymized mobile-device location data. The data are supplied through the TERAIN analytics platform and originates from a large opt-in panel of smart-device users who have consented to the collection of approximate and fine location signals via partner mobile applications. All data processing is fully anonymized and conducted in compliance with South Africa’s Protection of Personal Information Act (POPIA). No personally identifiable information is accessed, stored, or released at any stage of the data pipeline.

### 4.1 Temporal coverage and scale

The dataset covers a two-year period from January 2022 to December 2023. Raw mobility signals are captured at high temporal resolution and initially processed at the daily level. Analytical summaries are subsequently provided at monthly and annual scales, where appropriate. All descriptive statistics reported in this article are computed using annual aggregations to ensure temporal comparability across years. Across the two-year period, the dataset contains a total of **87,204,056 inferred mall visits**.

### 4.2 Mobility signal processing and visit detection

Raw location signals are generated through GPS, Wi-Fi, and cellular connections embedded in partner mobile applications. These raw “pings” are processed through a spatiotemporal clustering and dwell-time analysis pipeline to identify meaningful stops and movements. To convert raw movement traces into mall visits, Fetch Analytics applies a standard threshold-based approach widely used in mobility research: a device is counted as a mall visit only when it remains within a mall’s geographic boundary for a minimum duration, thereby excluding brief pass-bys and transient signal noise. Signal jitter and antenna triangulation effects are mitigated through clustering and smoothing procedures prior to visit inference. This process yields reliable estimates of foot traffic volumes and visits frequencies across malls and time periods [7].

### 4.3 Journey reconstruction and travel distance estimation

Following visit detection, journeys are reconstructed by linking identified mall visits to inferred home locations. Home locations are estimated based on repeated night-time stays over extended periods, a commonly applied method in mobility analytics. Travel distance is calculated as the straight-line (Euclidean) distance between the inferred home location and the mall location. While this measure does not reflect actual route distance, it provides a consistent and interpretable proxy for spatial accessibility and travel burden across visitors and malls.

### 4.4 Representativeness and calibration

Mobile-device data do not constitute a census of the population and may underrepresent individuals without smartphones or those who opt out of location services. To mitigate potential biases, Fetch Analytics applies post-stratification and calibration techniques using external demographic benchmarks, including census-based population distributions by age group, income proxy, and geographic area. These weighting procedures align observed device activity with known population structures at appropriate spatial scales, improving the representativeness of aggregated indicators while maintaining strict privacy safeguards.

### 5.5 Socio-demographic and psychographic variable generation

Socio-economic and demographic characteristics — such as income group, Living Standard Measure (LSM), education level, life-stage composition, and transport mode — are assigned by linking each device’s inferred home area to externally validated demographic and segmentation datasets. These attributes are not observed at the individual level but are probabilistically inferred based on area-level characteristics. Additional psychographic and life-stage segmentation variables are derived using standard classification frameworks commonly applied in market research and urban analytics. The released dataset includes both disaggregated indicators and higher-level aggregated measures, as summarized in Table 1.1 (Aggregated Indicators).

### 4.6 Data aggregation and release

All device-level information is aggregated to the mall–time level prior to release. The final dataset contains only aggregated statistics, including total visits, average travel distance, and the socio-economic composition of visitors by mall and year. This aggregation ensures user anonymity while providing a robust and transparent foundation for research in retail geography, urban mobility, applied statistics, and spatial economics.

### 4.7 Data quality considerations and limitations

As with all mobile-device–based mobility datasets, potential biases may arise from heterogeneity in network signal availability, device sampling rates, and user opt-in behavior. To mitigate these effects, the data provider applies signal-cleaning procedures, spatiotemporal clustering, and minimum dwell-time thresholds prior to visit inference, thereby reducing the influence of transient pings, antenna handoffs, and irregular sampling. In addition, post-stratification and calibration techniques based on external demographic benchmarks are used to align observed device activity with known population structures. Nevertheless, the dataset should be interpreted as an aggregated and probabilistic representation of shopping mobility rather than a complete census of individual movements. These limitations are common to large-scale location-intelligence data and should be considered when conducting fine-grained causal inference or individual-level interpretation.

## Limitations

• The data is limited to smartphone users, potentially excluding certain demographic groups.
• Income and LSM values are inferred from aggregated geospatial statistics rather than self-reported.
• The data does not capture in-mall shopping behaviour or spending habits.
• Potential biases may arise from network signal variations and device sampling rates.

## Ethics Statement

• No personally identifiable information (PII) is included in the dataset.
• This dataset was sourced free of charge with permission from Fetch Analytics.
• The dataset is readily available, free of charge, to be used by other researchers.
• Data collection complies with international privacy regulations and ethical standards.

## CRedit Author Statement

• Conceptualization: Koech Cheruiyot, Yewande Adewunmi, John Karuitha.
• Data Curation: Kalasipa Moenyane, Koech Cheruiyot, John Karuitha, Kelly Cohen.
• Methodology: John Karuitha, Prisca Simbanegavi.
• Analysis & Interpretation: John Karuitha, Yewande Adewunmi, Koech Cheruiyot.
• Writing – Original Draft: John Karuitha, Yewande Adewunmi, Koech Cheruiyot.
• Writing – Review & Editing: All authors.

## Declaration of Competing Interest

The authors declare that they have no known competing financial interests or personal relationships that could have appeared to influence the work reported in this paper.

## Data Availability

Mendeley DataDataset on Shopping Mall Visits in Johannesburg, South Africa (Original data).
